# Role of the Novel Hsp90 Co-Chaperones in Dynein Arms’ Preassembly

**DOI:** 10.3390/ijms20246174

**Published:** 2019-12-07

**Authors:** Hanna Fabczak, Anna Osinka

**Affiliations:** Laboratory of Cytoskeleton and Cilia Biology, Nencki Institute of Experimental Biology, Polish Academy of Sciences, 3 Pasteur Str., 02-093 Warsaw, Poland

**Keywords:** Hsp90, R2TP, DNAAFs, SPAG1, WDR92, ODA, IDA, dynein arm preassembly

## Abstract

The outer and inner dynein arms (ODAs and IDAs) are composed of multiple subunits including dynein heavy chains possessing a motor domain. These complex structures are preassembled in the cytoplasm before being transported to the cilia. The molecular mechanism(s) controlling dynein arms’ preassembly is poorly understood. Recent evidence suggests that canonical R2TP complex, an Hsp-90 co-chaperone, in cooperation with dynein axonemal assembly factors (DNAAFs), plays a crucial role in the preassembly of ODAs and IDAs. Here, we have summarized recent data concerning the identification of novel chaperone complexes and their role in dynein arms’ preassembly and their association with primary cilia dyskinesia (PCD), a human genetic disorder.

## 1. Introduction

Motile cilia are evolutionarily conserved, microtubule-based organelles present in numerous types of organisms from protists to mammals. These thin (approximately 0.2 µm in diameter) but long (usually up to 20 µm, and, in the case of some insect sperm flagella, more than 100 µm [1]) organelles jut from the apical cell surface and are crucial for the functioning and asymmetric arrangement of the major visceral body organs. In humans, motile cilia are usually assembled in large numbers by the epithelial cells lining the trachea, oviduct, ventricular system of the brain and the spinal cord. The coordinated planar beating of motile cilia generates movement that facilitates the clearing of mucus out of the airways, and supports transport of the ovum in the oviduct and circulation of cerebrospinal fluid in the brain ventricles. Single, long cilia, called flagella, enable movement of the sperm cells [2]. Specific motile cilia, so-called nodal cilia, are present in the embryonic node. Nodal cilia beat circularly, generating a leftward-directed fluid flow [3,4], and play a critical role in the generation of the left-right asymmetry of the arrangement of the major visceral body organs [5].

**Figure 1:**
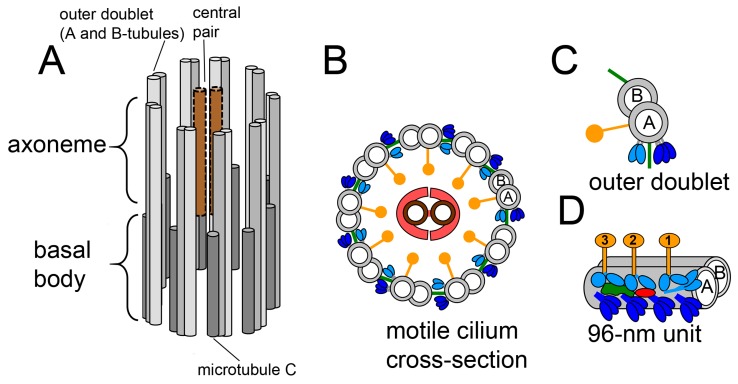
Schematic representation of motile cilia structure. **A**: Longitudinal view of the cilium. The nine peripheral doublet microtubules (gray) are a continuation of two out of three microtubules of the basal body microtubular triplets surrounding the central pair (brown); **B**: The cross-section of the axoneme from *Tetrahymena thermophila* shows nine doublet microtubules (A- and B-tubules, gray) surrounding a central pair of singlet microtubules (brown) with projection (orange). Attached to the doublet microtubules are the radial spokes (yellow), inner dynein arm (IDA, light blue), outer dynein arm (ODA, dark blue), nexin-dynein regulatory complex (NDRC, green) and modifier of the inner arms complex (MIA complex, red); **C**: The view of the doublet microtubule with attached macrocomplexes. (description as in B); **D**: The organization of macro-complexes within the 96-nm unit (description as in B) [7].

The scaffold of the motile cilium, the axoneme, is composed of nine outer microtubule doublets with docked multiprotein complexes and a pair of central microtubules with attached projections (9 + 2 structure). The outer doublet complexes, such as nexin-dynein regulatory complexes (N-DRC), radial spokes (RS1–RS3) and axonemal dynein arms, repeat periodically, forming a characteristic pattern along the entire cilium length with a basic, 96-nm unit. Each 96-nm repeat contains four ODAs, seven IDAs, three RSs, N-DRC, and other minor complexes [6]. Mammalian nodal cilia lack the central pair of microtubules, radial spokes, and IDAs (9 + 0 organization) [3] (Figure 1). 

Dynein arms facilitate the ATP-dependent microtubule sliding, which drives the ciliary movement [8]. Both outer dynein arms (ODAs) and inner dynein arms (IDAs) are large multi-subunit complexes that differ in their protein composition and motor properties. ODAs contain three (in Stramenopiles, Alveolata, and Rhizaria) or two (in metazoans and excavates) motor-domains containing dynein heavy chains (DHC) [9], two intermediate chains (IC1, IC2 in *Chlamydomonas* and DNAI1, DNAI2 in vertebrate) and about 10 light chains (LCs). ODAs are attached to the microtubules via ODA docking complexes (ODA-DCs) [10,11].

IDAs, present in one 96-nm unit, differ in their protein composition and likely function. Only IDA f/l1 contains two heavy chains, while the other IDAs (a,b,c,d,e,g) have one heavy chain, specific to each arm. IDA f/l1, in addition to two dynein heavy chains, different from chains of ODAs or/and monomeric IDAs, consists of at least seven additional subunits named, according to their size, intermediate chains (IC: IC140, IC138, and IC97) and light chains (LC: Tctex1, Tctex2b, LC7a, LC7b, and LC8) [10].

A growing number of studies indicate that the molecular mechanismcontrolling assembly of ODAs and IDAs, and their targeting to cilia, is evolutionarily conserved. Subunits of the dynein arms are synthesized, and dynein arm complexes are preassembled in the cytoplasm. The pre-assembled dynein arms are transported to cilia by intraflagellar transport (IFT) [12,13,14,15]. At least a dozen proteins known as axonemal dynein assembly factors (DNAAFs) participate in the preassembly of ODA and IDA [16,17,18]. Interestingly, DNAAFs were discovered during the search for the causative mutations in individuals affected by primary ciliary dyskinesia (PCD), generally an autosomal recessive disease that manifests by defective cilia/flagella motility. 

DNAAFs interact with and chaperone Hsp70 (heat shock protein 70), Hsp90 (heat shock protein 90), and Hsp90 co-chaperones, R2TP (RuvB-like protein 1 (RuvBL1), RuvB-like protein 2 (RuvBL2), RNA polymerase-associated protein 3 (RPAP3), and PIH1 domain-containing protein 1 (PIH1D1)) and R2TP-like complexes [16,19]. Here we have summarized recent advances in the identification of potential components of R2TP-like complexes and in understanding their role in dynein arms’ preassembly in several model organisms and in the etiology of the primary ciliary dyskinesia (PCD).

## 2. Hsp90 and Its Co-Chaperones

Hsp90 is a widespread molecular chaperone important for protecting cells from stress, such as high temperatures [20]. However, Hsp90 regulates many biological processes such as cell-cycle progression, telomere maintenance, apoptosis, mitotic signal transduction, vesicle-mediated transport, immunity, and targeted protein degradation [21]. Hsp90 functions in vivo as the core component of a dynamic set of multiprotein complexes, collaborating with a plethora of proteins or co-chaperones [22]. An important Hsp90 co-chaperone is an R2TP complex that assists Hsp90 in the assembly of large protein complexes (L7Ae ribonucleoproteins, U5 small nuclear ribonucleoprotein, RNA polymerase II, phosphatidylinositol-3-kinase-related proteins). The R2TP complex also participates in the pre-assembly of the dynein arms [23]. 

### 2.1. Composition of R2TP Complex 

**Figure 2:**
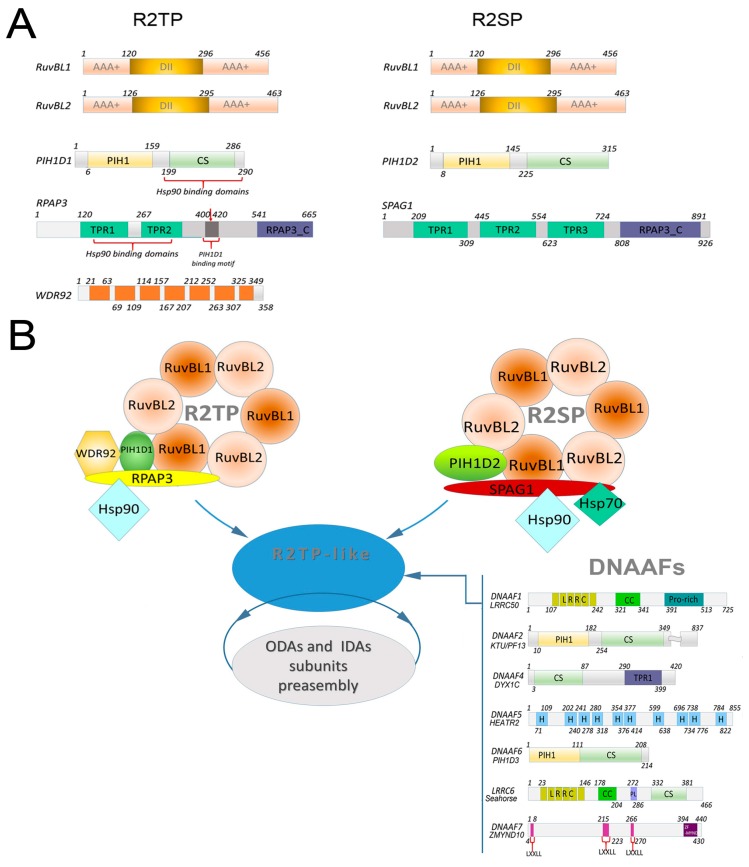
Proposed model of RT2P-like complex structure and function. **A:** Domain structure of human R2TP (RuvBL1, RuvBL2, PIH1D1, RPAP3, WDR92) and R2SP (RuvBL1, RuvBL2, SPAG1). **B:** Diagram illustrating the possible function of an RT2P-like complex that includes R2TP and/or R2SP and dynein axonemal assembly factors (DNAAFs) involved in the process of pre-assembly of axonal dynein arms. DNAAFs, together with chaperones (Hsp90, Hsp70), co-chaperons R2TP and R2SP, and axonemal dyneins form a cytoplasmic organelle-like structure called DynAPs (dynein axonemal particles) [27], for details, see Table 1. Domain organization: RPAP3 (RPAP3-Cter domain); TPR (tetratricopeptide repeat); PHI1 (protein interacting with heat shock protein 90); CS (CHORD-containing proteins and SGT1); WD (WD40 repeat); LRRC (Leucine-Rich Repeat-Containing); H (HEAT Repeat-Containing); CC (coiled-coil domain); LxxLL (Leu-Xaa-Xaa-Leu-Leu) motif; ZF-MYND (Mynd-type zinc finger domain).

The R2TP complex was discovered in *Saccharomyces cerevisiae* as an Hsp90 co-chaperone [24]. In humans, the canonical R2TP complex consists of a hexamer composed of two AAA + ATPases related proteins, RuvBL1/Pontin and RuvBL2/Reptin, and a heterodimer composed of RPAP3 and PIH1D1 (Figure 2). The RPAP3–PIH1D1 heterodimer is an integral and specific component of R2TP and likely regulates the enzymatic activity of RuvBL1 and RuvBL2 [25]. The RuvBL1 and RuvBL2 AAA ATPases, due to their enzymatic activity, form the catalytic component of the R2TP complex, probably acting not only as co-chaperone but also as a chaperone [26].

The N-terminal region of PIH1D1 (Figure 2) has the conserved PIH1 domain (protein interacting with heat shock protein 90) known to mediate the binding of proteins containing a specific motif phosphorylated by casein kinase 2 (CK2) [40,41,42]. The C-terminal region of PIH1D1 has a CS domain (CHORD-containing proteins and SGT1), also present in other Hsp90 co-chaperones (such as p23) [43]. The C-terminal domain (Pih1D1-C-ter), comprising the CS domain, mediates direct interactions between PIH1D1 and RPAP3 and between RuvBL1 and RuvBL2 [42]. RPAP3 (Figure 2) possesses a C-terminal domain of unknown function (RPAP3-C-ter) and two tandem N-terminal TPRs (tetratricopeptide repeats) domains. The TPR domains enable binding of RPAP3 to Hsp70 [44,45] and Hsp90 (more precisely, to the C-terminal fragment of Hsp90 containing a conserved MEEVD motif/sequence) (Figure 2) [19,40,46]. 

### 2.2. R2TP Complexes in the Dynein Arm Assembly 

The first indications that RuvBL1 and RuvBL2 could play a role in the formation of motile cilia came from studies in *Chlamydomonas*. The genome-wide transcriptional analyses revealed that RuvBL1 and RuvBL2 are strongly upregulated during the regeneration of *Chlamydomonas* flagella [47]. Later, using immunofluorescence analyses it was shown that, in embryos of *Danio rerio*, RuvBL1 and RuvBL2 is present in cells assembling motile cilia (extra-renal tissues, pronephric tubule cells, Kupffer’s vesicle, the neural tube) [15]. *Danio rerio* mutants with loss-of-function mutation in genes encoding RuvBL1 or RuvBL2 have phenotypes typically observed in embryos with defects in the motile cilia, including a curved body shape and kidney cysts. Although the levels of transcription in many components that build the dynein arms were unchanged [15,48], the transmission electron microscopy (TEM) analysis showed that, in both mutants, cilia had a reduced number of ODAs and IDAs [15]. Immunofluorescence analysis of mouse ependymal and oviduct epithelial multiciliated cells, as well as studies of Kupffer’s vesicle and pronephric ciliated cells in zebrafish, showed that RuvBL1 was present in the cytoplasm [15,49] and in the nucleus [49]. Contradictory to these data, using a non-commercial antibody, it was shown that in *Chlamydomonas* RuvBL1 was also present in flagella [50]. 

In transgenic RuvBL1^frt-fl^ mice disruption of ciliary integrity was observed, causing renal disease and hydrocephalus [49]. In mice, loss-of-function mutations of RuvBL1 is lethal at an early embryonic stage, but deletion of the RuvBL1 only in premeiotic male germ cells causes male infertility [15]. A reduction and/or lack of DNAH9 (Dynein heavy chain), DNAI2 (Dynein intermediate chain), and DNALI1 (light intermediate chain-1) in sperm flagella and cilia of multiciliated cells suggest the possible involvement of RuvBL1 in dynein arms’ preassembly or stability [15,49]. Interestingly, the co-immunoprecipitation analyses (from testis of mutant mice) revealed that when RubBL1 was mutated, both RuvBL1-RuvBL2-Hsp90 and DNAI1-DNAI2 complexes failed to form. These data can point to the role of RuvBL1 and RuvBL2 as co-chaperones in the folding and stabilization of DNAI1-DNAI2 complex (Table 2) [15]. To date, mutations in RuvBL1 or RuvBL2 have not been associated with PCD in humans, likely because these proteins function as co-chaperones for the assembly of diverse protein complexes and their mutations would cause death during early development, as was observed in mice [15]. 

Similarly, the analyses of *Chlamydomonas* and *Danio rerio* mutants suggested the involvement of PIH1D1 in dynein arms’ assembly. Comparative studies of *Danio rerio* WT and pih1d1_null mutant revealed the abnormal motility of sperm flagella and Kupffer’s vesicle cilia caused by the loss of outer and inner dynein arms. As confirmed by cryo-electron tomography analyses of cilia assembled by the *Danio rerio* mutant, PIH1D1 is necessary for the proper assembly of ODA (DNAI1 was not detected using immunofluorescence) and IDA c (Table 2) [52]. Accordingly, the *Chlamadymonas ida10* mutants lacking MOT48, an ortholog of PIH1D1 [51], are either immotile or move significantly slower. The phenotypic changes were caused by the reduction in the number of IDAs b and c, while IDAs f/I1 and g were almost unaffected or only slightly reduced, similarly to ODAs [51]. The biochemical studies revealed that MOT48 interacts with RuvBL1, RuvBL2, RPAP3, and DNAAF4/DYX1C (Table 1) [53]. 

The WD repeat-containing protein 92 (WDR92) has been reported to associate with the R2TP complex (Figure 2) [59,60]. Recently, the participation of WDR92 and the canonical R2TP complex in the pre-assembly of dynein arms has been revealed [53,54,55,61]. The comparative genomic studies demonstrate that *WDR92* genes are highly conserved in organisms with motile cilia [54,61]. The initial evidence that *WDR92* associates with ciliary motility proteins was provided by studies in planarian *Schmidtea mediterranea*. It was shown that animals deficient in WDR92 still assembled in cilia of normal length, and in similar numbers to WT, but ciliary beat frequency was reduced. TEM analysis revealed that many cilia had defects in their architecture, including partial loss of the outer dynein arms [61]. WDR92 is also required for the assembly of ODAs and IDAs in *Drosophila* and *Chlamydomonas* (Table 2). *Drosophila melanogaster* has only two cell types bearing motile cilia: the sperm flagellum and the ciliated proprioceptive chordotonal sensory neurons [55]. Mutations in *WDR92* result in male infertility and auditory deficiency. Cross-sections of the cilia of *WDR92 Drosophila* mutants showed an absence of ODAs and IDAs [55]. The *Chlamydomonas WRD92* mutant assembles very short cilia lacking inner and outer dynein arms. Moreover, the level of dynein arm heavy chains in the cytoplasm is low [53,54]. Data obtained during the comparative mass spectrometry analysis of wild type and *Drosophila* mutant sperm flagella [55], and analyses of the WDR92 *Chlamydomonas* phenotype [54], suggest that WDR92 is required for the stabilization of the folded dynein heavy chains (Table 2). Interestingly, in *Drosophila* WDR92 mutants the level of DNAAF2/KTU/PF13 and DNAAF4 was elevated, suggesting that DNAAF2 and DNAAF4 could form an R2TP-like co-chaperone complex for an early HC assembly step [55]. Moreover, in S2 *Drosophila* cells, WDR92 co-immunoprecipitates with several proteins, including RPAP3, PIH1D1, RuvBL1, RuvBL2, DNAAF1/LRRC50/ODA7 and SPAG1 (Table 1) [55]. Similarly, immunoprecipitation followed by mass spectrometry revealed that in *Chlamydomonas*, WDR92 directly or indirectly interacts with inner dynein arm heavy chains, RuvBL1, and multiple DNAAFs, including RPAP3, MOT48 (*Chlamydomonas* orthologue of PIH1D1), ODA7 (*Chlamydomonas* orthologue of DNAAF1), and DNAF4 (Table 1) [53]. In mammalian and *Drosophila* cells, WDR92 binds to RPAP3, potentially through the RPAP3 C-terminal domain [55,62]. 

### 2.3. Composition of R2SP Complex and Function in Dynein Arm Preassembly

The R2SP (RuvBL1-RuvBL2-SPAG1-PIH1D2) complex is related to the R2TP complex, with RPAP3 and PIH1D1 adaptors being replaced by SPAG1 (sperm associated antigen 1) and PIH1D2, respectively [63] (Figure 2). SPAG1 contains three TPR domains in its N-terminal end and RPAP3-C-ter domain at its C-terminus (Figure 2). Moreover, SPAG1 also interacts with Hsp90 and Hsp70 [63].

Data from various organisms indicate that SPAG1 is involved in the pre-assembly of dynein arms [28,63,64]. Knockdown of SPAG1 in *Danio rerio* caused a phenotype typically observed when motile cilia are dysfunctional (dorsal body axis curvature, and hydrocephalus [28]). Sperm cells of the *Drosophila* mutant depleted of CG18472, an orthologue of SPAG1, were immotile. Moreover, the TEM analysis of the chordotonal sensory neuron cilium revealed a loss of dynein arms (ODAs, IDAs) (Table 2) [55]. In humans, mutations in SPAG1 cause primary ciliary dyskinesia (CILD28) [28].

SPAG1, like other proteins with TPR domains, (Table 1) mediates the delivery of clients from Hsp70 to Hsp90, thanks to simultaneous anchoring of the chaperones via EEVD residues within Hsp70 and Hsp90 C-terminal end to the designed TPR domains of SPAG1 [64]. SPAG1, besides RuvBL1, RuvBL2, and PIH1D2, interacts (possibly indirectly) with DNAAF5/HEATR2 (HEAT Repeat-Containing) and DNAAF2 (Table 1), and co-localizes with them in DynAPs [27,65]. Recent studies on ciliogenesis in human primary airway epithelial cells have shown that DNAAF5, SPAG1, and DNAAF2 undergo transcription during the early stages of airway epithelial cell differentiation. The analyses of the epithelial respiratory cells obtained from PCD patients during biopsies have shown that mutations in DNAAF5 do not disrupt direct interaction with SPAG1, but cause the accumulation of large DNAAF5–SPAG1-DNAAF2 aggregates and degradation of all three proteins [65]. 

On the other hand, preassembly genes such as DNAAF1, DNAAF3, DNAAF4, LRRC6, DNAAF6/PIH1D3/TWISTER/TWI, and DNAAF7/ZMYND10, were expressed at a later stage of cell differentiation, similar to dynein DNAI1 [65]. Based on these data, it can be assumed that R2SP is involved in the early phase of the formation of dynein arms when SPAG1 binds to DNAAF2. However, the R2SP complex can interact with the late preassembly proteins, e.g., DNAAF4 and DNAAF6 [30,33] and WDR92, as was shown in human cells [66] and later confirmed in *Drosophila* [55]. It is likely that WDR92 contributes to cooperation between R2SP and R2TP [55]. Biochemical analyses of *Drosophila* testis showed that SPAG1 binds to Dhc98D (DNAH10), a heavy chain inner arm dynein also associated with WDR92 [55]. In the presence of overexpressed RuvBL1 and RuvBL2, the association of SPAG1 with WDR92 was enhanced in S2 *Drosophila* cells. Such observations favor the hypothesis that WDR92-SPAG1 may form part of a conserved co-chaperone complex [55]. 

The PIH1D2 (Figure 2) protein plays an important role in the formation of the R2SP complex by facilitating the interaction between SPAG1 and RuvBL1-RuvBL2 (Table 1) [63]. Moreover, recent data indicate that PIH1D2 is also involved in dynein arms’ assembly. Cilia in Kupffer’s vesicle and sperm cell flagella of *Danio rerio* pih1d2-null mutant are either immotile or move irregularly. Accordingly, cryo-ET and biochemical analyses revealed that the PIH1D2 protein is required for the assembly of ODA and IDA b, c, and e subunits (Table 2) [52].

### 2.4. DNAAFs and Their Function in Dynein Arm Assembly

As mentioned above, the identification of the causative mutations in the individuals affected by PCD, with characteristic loss of dynein arms without changes in the genes encoding dynein arm components, led to the discovery of a number of cytoplasmic proteins called DNAAFs that participate in dynein arms’ preassembly.

Although the ciliary ultrastructural outcome of the mutations in DNAAFs is well known, the molecular mechanisms behind their activity, and data concerning DNAAFs’ partners and interactions between DNAAFs and chaperones/co-chaperones, are fragmentary. Below, we briefly summarize the current state of knowledge. 

Proteins that function as DNAAFs contain different domains (e.g., TPR, PIH1, CS, LRRC) (Figure 2) that enable interactions with other proteins and complex formation. The genomes of the vertebrates encode four proteins with a PIH1 domain at the N-terminal end and CS domain in the C-terminal fragment: PIH1D1, PIH1D2, both described above, which form the canonical R2TP and R2SP complexes, respectively [19], and dynein assembly factors, DNAAF2 and DNAAF6 [15,63,67]. 

DNAAF2/KTU/PF13 was the first protein identified as a DNAAF (Figure 2, Table 1) [16]. The presence of PIH1 and CS domains suggest that DNAAF2 could potentially form a complex with RuvBL1 and RuvBL2. However, such interactions in vivo were not confirmed at least in *Chlamydomonas* [53]. On the other hand, DNAAF2 interacts with DNAAF4 [30,53], which can facilitate complex formation between DNAAF2–DNAAF4 and Hsp90 [34,63] and Hsp70 [16]. DNAAF2 is required for ODA and IDA heavy chain stability, possibly by assisting the assembly of the heavy chains with other subunits (Table 2). The flagella in *Chlamydomonas* mutants lacking DNAAF2 are completely paralyzed [16]. Studies of the *Danio rerio* mutant lacking DNAAF2 revealed normal sperm motility, but the motility of cilia in Kupffer’s vesicle was irregular, or exhibited a slower rotation, caused by the loss of dynein arm ODA, and IDA b, c, and e subtypes (Table 2) [52]. 

Genetic, immunocytochemical and TEM studies performed on fish medaka (*Oryzias latipes*) and human respiratory cells have shown that, in the absence or mutation of DNAAF2, cilia are assembled normally, but both the outer and inner dynein arms are missing [16]. In humans, mutations in the gene encoding DNAAF2 cause PCD (CILD10) [16].

With the exception of Olcese and co-workers [34], in several other studies it was suggested that the PIH1 domain is present in the N-terminal fragment of DNAAF6/PIH1D3 [33,52,63,67] (Figure 2). DNAAF6 also contains a CS domain at its C-terminus and, as confirmed by co-immunoprecipitation, analysis can form a complex (interacting directly or indirectly) with Hsp70 and/or Hsp90 [34,67]. In humans, mutations in the X-linked DNAAF6/PIH1D3 causes PCD (CILD36), due to the defects in both the outer and inner dynein arms [33,34]. In contrast to the human genome encoding a single *PIH1D3* gene, two *Pih1d3* genes are present in mice genome, one located on chromosomes 1 (*Pih1d3a*, 4930521A18Rik) and one on chromosome X (*Pih1d3b*, E230019M04Rik). Pih1d3a and Pih1d3b proteins are 91% identical, with differences mainly within the unstructured N-terminal end. Pih1d3a is present exclusively in the testis. Mice with knocked out *Pih1d3a* produce sperm cells with immotile flagella due to dynein arm defects, while motile cilia in other organs are unaffected. Immunochemical analysis revealed that the levels of DNAH9/HCβ, DNAIC1/IC1, and DNAIC2/IC2 (components of ODAs), and DNAH7/HC (a component of IDAs) were reduced in knock-out sperm compared with those of WT mice [67]. The X-linked *Pih1d3b* is expressed in mice lung, brain, and oviduct. The *Pih1d3b* cannot rescue the sperm flagella defect arising from the loss of *Pih1d3a*. In *Danio rerio*, mutants lacking DNAAF6 sperm flagella and Kupffer’s vesicle cilia are immotile. Cryo-electron tomography showed that the mutated axoneme lacks ODA, IDA c, g, and d (Table 2) [52]. Interestingly, mutations in TWI1, a *Chlamydomonas* ortholog of DNAAF6, do not affect flagella motility and cells’ swimming, suggesting that TWI1 is most likely not involved in dynein arm assembly in this green algae [53]. On the other hand, like other proteins involved in the dynein arms’ assembly, TWI1 is strongly upregulated upon deflagellation [47]. 

Some DNAAFs, in addition to a CS domain, also have TRP domains that enable direct binding to Hsp70 and/or Hsp90. DNAAF4/DYX1C (Figure 2) has three TRP domains and it can form a DNAAF2-DNAAF4-Hsp90 co-chaperone complex (Table 1), and, thus, can link the assembly of axonemal dynein to Hsp90 [30,53,68]. Tarkar and co-workers [30] showed that DNAAF4 deficiency in humans, mice, and zebrafish results in defects in the motile cilia, caused by deficient dynein arm transport or assembly. The deletion of DNAAF4 in mice [30] and mutations in humans [30] cause symptoms characteristic of PCD (chronic airway disease, laterality defects, and male infertility) (CILD25). Similar phenotypic changes typical for motile ciliary dysfunction (laterality and ciliary motility defects) were observed after a reduction in the expression of DNAAF4 in zebrafish [30]. Ultrastructural and immunofluorescence analysis of motile cilia assembled by the respiratory cells in mice and humans revealed disturbed ODA and IDA organization. The components of ODAs (DNAH5, DNAH9, and DNAI2) and IDAs (DNALI1) were absent or significantly reduced (Table 2) [30] in these cilia. 

Both RuvBL1 and RuvBL2 can also interact with DNAAF1/LRRC50/ODA7 (Leucine-Rich Repeat-Containing 50) (Figure 2, Table 1) [56]. Mutations of DNAAF1/ODA7 in *Chlamydomonas* reduce flagella beat frequency and a block outer dynein arm assembly [13,68]. The biochemical and immunofluorescence analyses indicate that DNAAF1/ODA7 and its orthologue in *Trypanosoma brucei* similar to other a DNAAFs, is localized in the cytoplasm [17,29]. DNAAF1 over-expressed in ciliated hTERT-RPE1 cells co-localized with IFT88 at the base of the primary cilium and RuvBL1 knockdown caused disruption of this co-localization [56]. In humans, cilia of the respiratory cells obtained from the patients carrying a DNAAF1 mutation (CILD13), lacked ODAs and IDAs subunits (DNAH5, DNAH9, DNAI2 and DNALI1) (Table 2) [29,57]. Interestingly, in humans the mutation in DNAAF1 was also associated with neural tube defects (DNAAF1 seems to be essential for the final step of neural tube closure) [69]. Similar to humans, mutations of DNAAF1 in mice and zebrafish cause changes typical of PCD in these species [56]. The biochemical analysis revealed that several heat shock proteins, including Hsp90, were more strongly associated with the mutanted DNAAF1 protein than the wild-type protein, which may be evidence of a possible instability of the mutated protein. While both RuvBL1 and RuvBL2 proteins interacted with wild-type DNAAF1, interaction with RuvBL1 was reduced in the case of the DNAAF1 mutant [56]. 

LRRC6/Seahorse (Leucine-Rich Repeat-Containing 6) (Figure 2) is another DNAAFs whose defects cause primary ciliary dyskinesia (CILD19) [37,38,39]. LRRC6, containing several LRR repeats is another protein expressed specifically in cells with motile cilia, including the nodal, tracheal, and sperm cells in mice [39]. LRRC6, in addition to six LRR repeats at the N-terminus, also contains a CS domain at the C-terminus [48]. The CS domain is likely essential for the direct interaction between LRRC6 and RuvBL2 [35,37,48] or DNAAF7 [35] (Table 1). In mutant mice and individuals affected with PCD lacking LRRC6 the length of cilia was normal, but nodal and typical motile cilia were immotile due to the loss of IDAs and ODAs [37,38,39] (Table 2). Probably, the lack of IDAs and ODAs in the axoneme of the mutant lacking LRRC6 was caused by defective transport of the dynein arm to the cilia [39]. 

DNAAF7/ZMYD10 (zinc finger MYND-type-containing 10) (Figure 2) is a cytoplasmic protein possessing Mynd-type zinc finger domain (myeloid, nervy, and DEAF-1) at its C-terminus that engages in protein-protein interaction [70]. DNAAF7 is highly expressed in cells assembling motile cilia, compared with nonciliated cells [36,71]. Previous studies have established a strong genetic link between loss or mutation of DNAAF7 and a combined outer and inner dynein arms defect in model organisms (fly, medaka, *Xenopus*, mouse) and humans (PCD, CILD22) [35,36,72,73]. DNAAF7 interacts with RuvBL2 [58] and LRRC6 [35,58,74]. It was shown that the C-terminal fragment of DNAAF7 and the CS domain of LRRC6 were necessary for this interaction [35]. DNAAF7 also forms complexes with C21ORF59 and DNAAF4, IDAs’ dynein light chain, and components of ODAs such as TCTEX1D1 (Tctex1 domain-containing D1) and DNAI1 (Table 2) [58]. Recently, Mali and co-workers [74] revealed that, in murine cells, DNAAF7 can form a novel chaperone complex comprising DNAAF7, FKBP8, and Hsp90, which can take part in the maturation of dynein heavy chains. 

In addition to the DNAAFs proteins described above, several other DNAAF proteins were identified: DNAAF3/C19orf51 (CILD2) [17,75], and CFAP300/C11orf70 (CILD38) [76,77,78], CFAP298/C21orf59/FBB18/KURLY (CILD26) [79]. However, until now their partner proteins and potential interactions with co-chaperones to form R2TP-like complexes were not described.

## 3. Conclusions and Perspective

The mechanism responsible for the cytoplasmic preassembly of the dynein arms components, the loading of these complexes onto the IFT system and their delivery and docking to the axoneme is still only fragmentarily understood. Although, in recent years, numerous proteins involved in the assembly of ODA and IDA have been identified, the still enigmatic interactions between chaperones, co-chaperones, DNAAFs and dynein arms subunits in this multi-step and complex processes are not clear. Moreover, to some extent, distinct mechanisms involved in the assembly of individual IDA and ODA components further impede understanding of these processes. In addition, it should be emphasized that impairment of dynein arms pre-assembly is not due to abnormalities in the level of transcription and/or translation, but the lower stability of individual ODA or IDA proteins resulting from the lack or dysfunction of R2TP, and/or R2SP and/or R2TP-like complexes. Also, the incomplete understanding of the mechanism of dynein arm folding and the role of co-chaperone complexes is partly due to methodological limitations. In the overwhelming majority of studies, the lack or lower level of individual dynein arms is determined by immunofluorescence labelling using antibodies that recognize individual proteins. Unfortunately, the availability of antibodies is limited to only few proteins, and more precise methods based on cryo-electron microscopy are only now being introduced. However, a better understanding of the mechanism of dynein arm preassembly is necessary and urgent. In humans, an improperly carried out preassembly of dynein arms results in primary ciliary dyskinesia (MIM:244400). PCD is a rare disease, affecting 1 in 10,000–15,000 live births [80,81]. The hallmark phenotype of PCD includes chronic sinusitis, recurring respiratory tract infections, infertility, situs inversus, and, rarely, hydrocephalus [82,83,84]. Moreover, around 50% of individuals affected with PCD suffer from disorders of laterality (situs inversus), also known as Kartagener syndrome, which is due to the failure of nodal cilia function and is often associated with cardiac development disorders [56,85]. A proper diagnosis of PCD is still challenging, especially when patients manifest non-specific signs and symptoms. This begs the question of how to help solve this problem. The identification of new PCD genes whose mutations may cause phenotypic changes significantly increases patients’ chances for proper diagnosis. The implementation of genetic and molecular diagnosis of PCD is a necessity, regardless of whether it is used as a confirmation of clinical diagnosis or when PCD is suspected. Unfortunately, hopes of the implementation of PCD gene therapy, which could help patients suffering from this disease, are still rather distant taking into account the current stage of knowledge.

## Figures and Tables

**Table 1:** List of proteins forming R2TP and other complexes involved in the preassembly of axonemal dynein arms.

Human Protein	Aliases	Protein Accession Number	Protein Size (kDa)	Localization in Cell	Domains	* Chaperone, Co-Chaprone and DNAAFs Interaction	PCD
RuvBL1,	Pontin	Q9Y265	50	DynAPs		Hsp90, RuvBL2, PIH1D1, RPAP3, WDR92, SPAG1	_
RuvBL2	Reptin	Q9Y230	51	DynAPs		RuvBL1, PIH1D1, RPAP3, WDR92	_
RPAP3	HSpagh	Q9H6T3	75	_	TPR- RPAP3-C-ter	Hsp70, Hsp90, RuvBL1, RuvBL2, PIH1D1, WDR92,	
PIH1D1		Q9NWS0	32	_	PIH1, CS	Hsp90, RuvBL1, RuvBL2; RPAP3, WDR92, DNAAF4	_
PIH1D2		Q8WWB5	39.4	_	PIH1, CS	RuvBL1; RuvBL2, SPAG1	_
WDR92	Monad	Q96MX6	39.7	DynAPs	WD40	RuvBL1, RuvBL2; RPAP3, PIH1D1, DNAAF1, DNAAF4, SPAG1	
SPAG1		Q07617	103.6	DynAPs	TPR, RPAP3-C-ter	Hsp70, Hsp90, RuvBL1, RuvBL2, PIH1D2, WDR92, DNAAF2, DNAAF4, DNAAF5, DNAAF6	MIM:615505 [28]
DNAAF1	LRRC50/ ODA7	Q8NEP3	40	cytoplasm	LRR	Hsp90, RuvBL1, RuvBL2,	MIM:613193 [29]
DNAAF2	KTU/ PF13	Q9NVR5	91	DynAPs	PIH1, CS	HSP70, HSP90, RuvBL1, RuvBL2, DNAAF4	MIM:612518 [16]
DNAAF4	DYX1C1	Q86X45	48.5	DynAPs	TPR, CS	Hsp70, Hsp90, DNAAF4 RuvBL1, RuvBL2 DNAAF2	MIM:615482 [30]
DNAAF5	HEATR2	Q86Y56	93.5	DynAPs	HEAT_type_2	DNAAF2, SPAG1,	MIM:614874 [31,32]
DNAAF6	PIH1D3	Q9NQM4	24	DynAPs	PIH1, CS	Hsp70, Hsp90, DNAAF1, DNAAF2, DNAAF3	CILD36 [33,34]
DNAAF7	ZMYND10	O75800	50	DynAPs	Znf_MYND	Hsp90, Hsc70 RuvBL2, LRRC6, DNAAF4,	MIM:615444 [35,36]
LRRC6	Seahorse	Q86X45	54	DynAPs	LRR, CS	RuvBL1, RuvBL2 DNAAF4,	MIM:614935 [37,38,39]

TPR domain (tetratricopeptide repeat), CS domain (CHORD-containing proteins and SGT1), LRRC (Leucine-Rich Repeat-Containing), PIH1 domain (protein interacting with heat shock protein 90), WD40 repeat protein, RPAP3-Cter domain. MIM (Mendelian Inheritance in Man, Phenotype MIM number; https://omim.org/). * Summary of main experimental data from biochemical analysis (e.g. co-immunoprecipitation, yeast two-hybrid system, protein-protein cross-link assays, GST-pulldown) in different model organisms.

**Table 2:** Summary of the experimental data showing which component of ODA and/or IDA are missing in cilia assembled by the cells with mutated co-chaperone or DNAAFs.

	*Chlamydomonas*		*Danio rerio*		Mouse		Humans	
	ODA	IDA	ODA	IDA	ODA	IDA	ODA	IDA
RuvBL1-RuvBL2					DNAH9, DNAI2 [15,49]	DNALI1 [49]		
PIH1D1/MOT48		IDA b, c, [51]	DNAI1 [52]	IDAc [52]				
PIH1D2			DNAH8, DNAI1 [52]	IDA b,c,e [52]				
WDR92	αHC, βHC, ϒHC, IC/LC [53,54]	DHC5, DHC8, DHC9 [53]	DNAH17, DNAH8 DNAI1-DNAI2, DNAL4 [55]	DNAH12, DNAH10, Centrin [55]				
SPAG1							DNAH5, DNAI1 [28]	DNALI1 [28]
DNAAF1/ODA7	HCα, IC70, IC78 [13]	ID1 [13]					DNAH5, DNAH9, DNAI2 [56,57]	DNALI1 [56,57]
DNAAF2	HCα, HCβ, HCϒ, [16]	HC9 [16]	DNAH8 DNAI1 [52]	IDA b,c,e [52]			DNAH5, DNAH9, DNAI2 [16]	DNALI1 [16]
DNAAF4					DNAH5, DNAH9, DNAI2 [30]	DNALI1 [30]	DNAH5 [30]	DNALI1 [30]
DNAAF5		DIC2/IC78 [32]	DNALI1 [31]				DNAH5 [31]	DNALI1 [31]
DNAAF6			DNAH8, DNAI1 [53]	IDA c,d,g [52]	DNAH5, DNAI1, [34]	DNALI1 [34]		
DNAAF7					DNAH5, DNAI2 [58]	TCTEX1D1 [58]	DNAH5 [35]	DNAL1 [35]
LRRC6							DNAI1, DNAI2 [37,38]	DNAH7 DNALI2 [37,38]
